# Supplementary figures and images for: Susceptibility profile of Anopheles and target site resistance mechanism against organophosphates in Cameroon
Source: PLoS One. 2025 May 22;20(5):e0321825. doi: 10.1371/journal.pone.0321825 (PMC12097638; doi:10.1371/journal.pone.0321825)

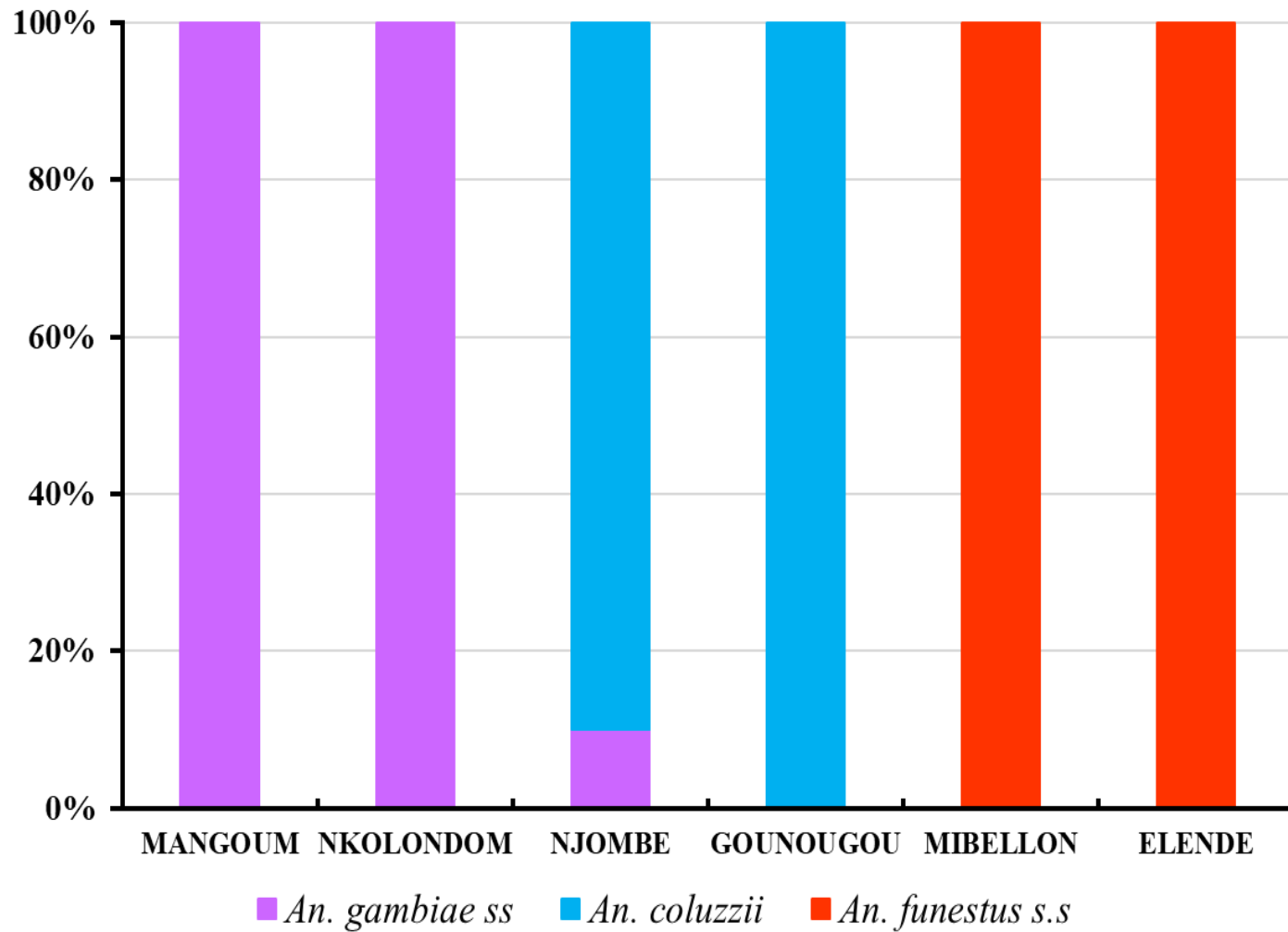

**S1 Fig:** species identification according to sampling site

Supplement: S1 Fig — (PDF) [file pone.0321825.s001.pdf]

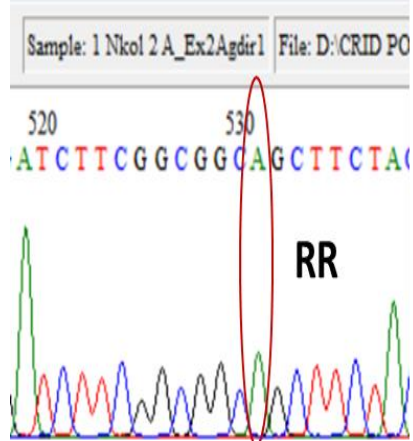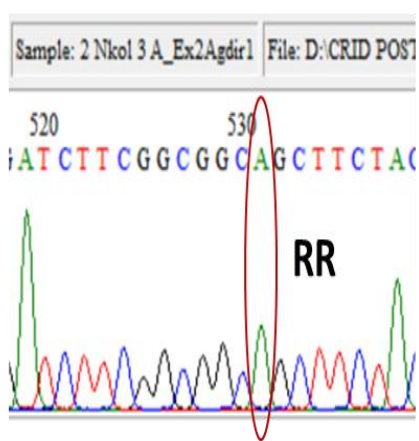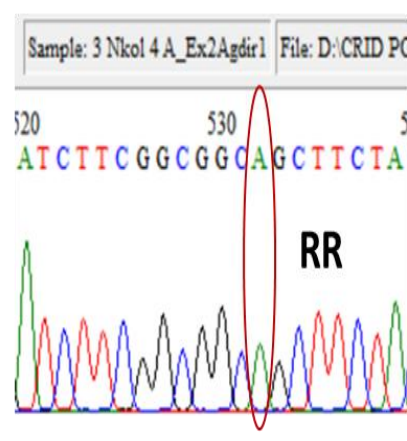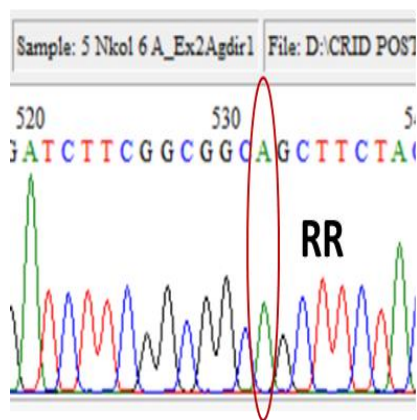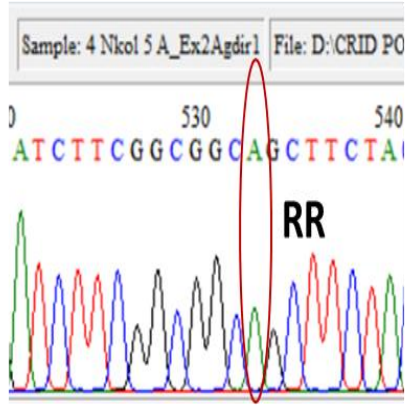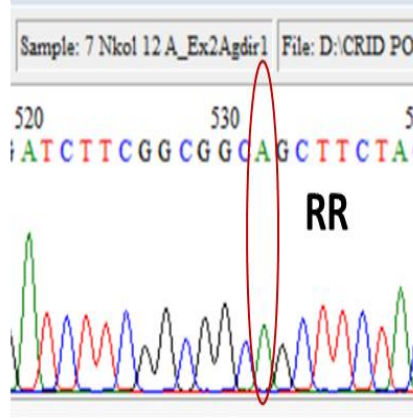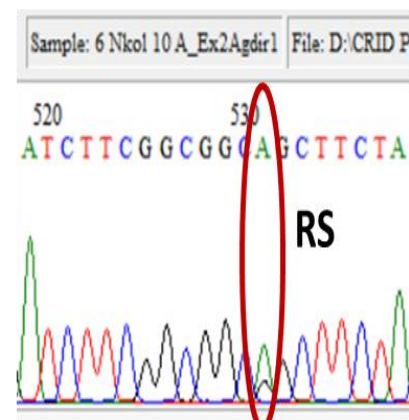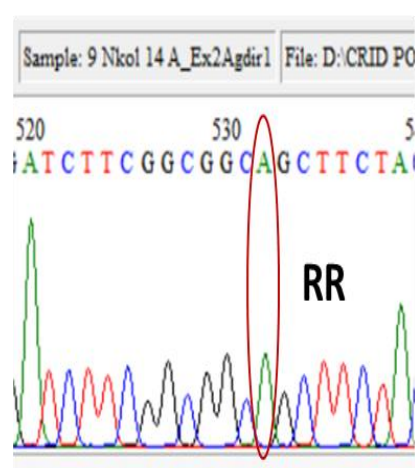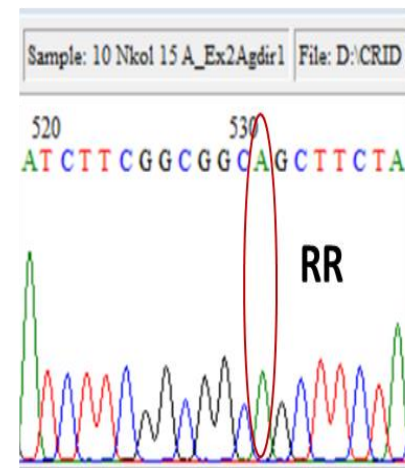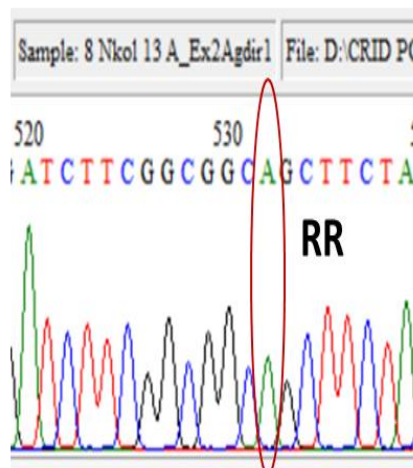

Supplement: S2 Fig — The figure highlights the presence of two peaks in one individual (the only one on the right) that was considered homozygote resistant with the TaqMan genotyping. (PDF) [file pone.0321825.s002.pdf]

## Dual Color Scatter Plot

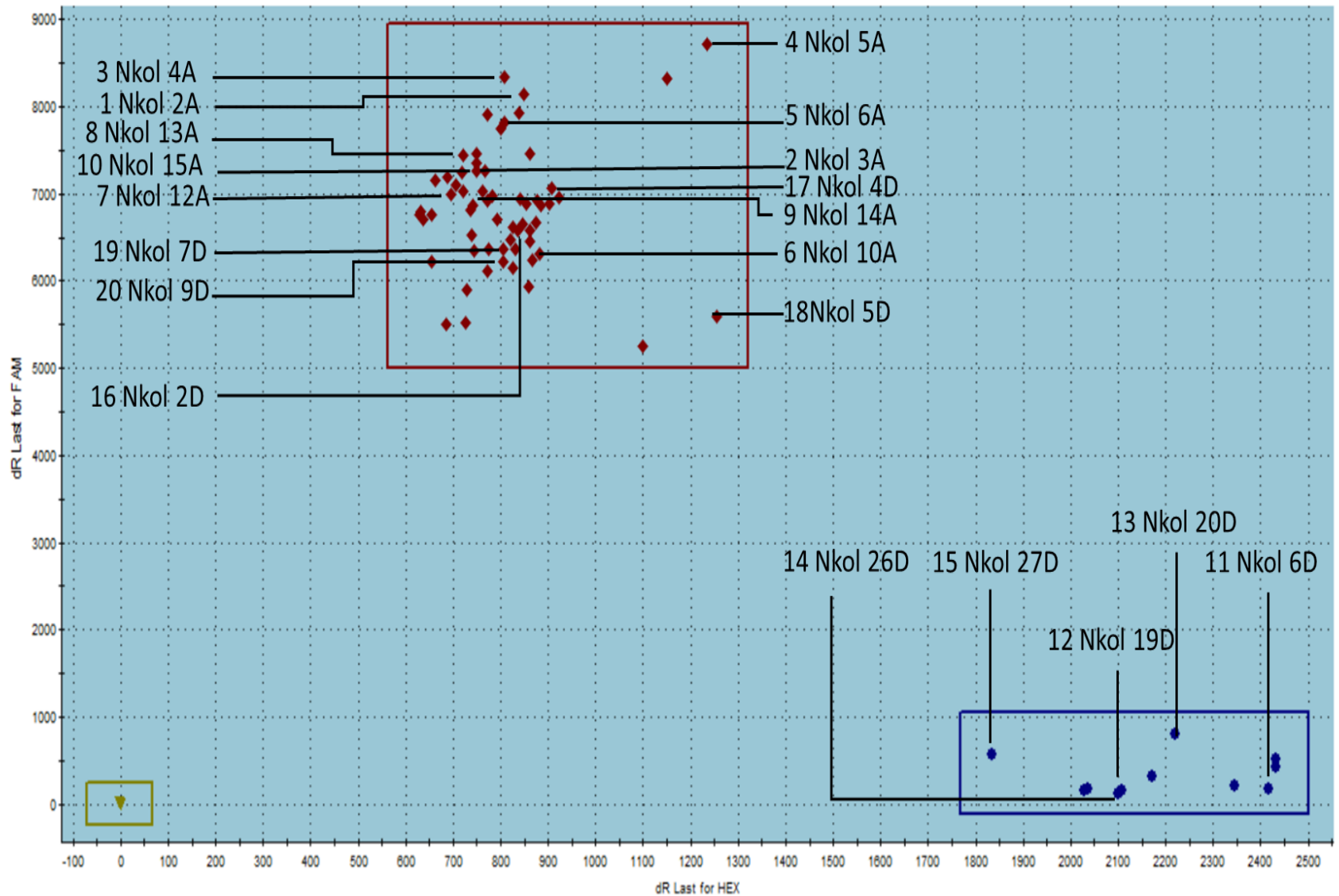

Supplement: S4 Fig — Two plots are visible, the one in red for “RR” mosquitoes and the one in blue for “SS” mosquitoes, no heterozygote was found. The selected samples for sequencing are identified with coding names. In the codes, A stands for Alive, and D for Dead phenotypes. (PDF) [file pone.0321825.s004.pdf]

(a)

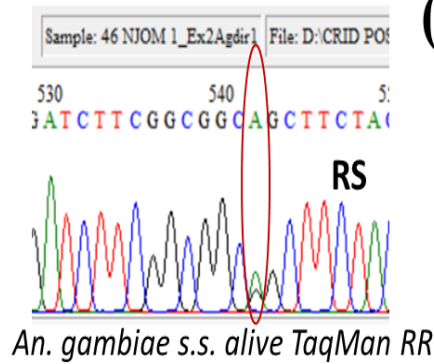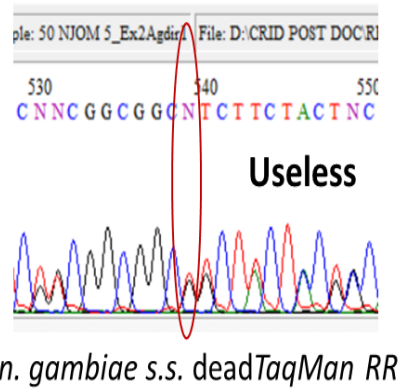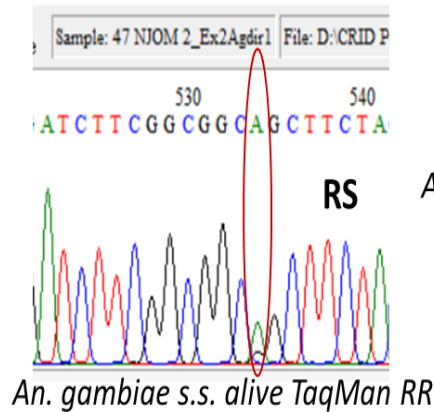

(b)

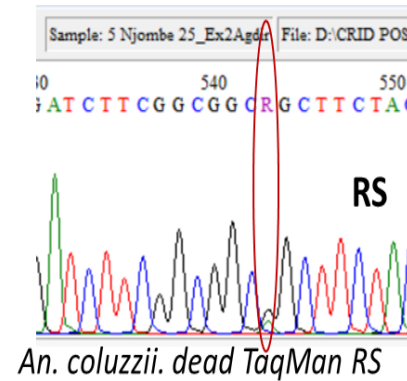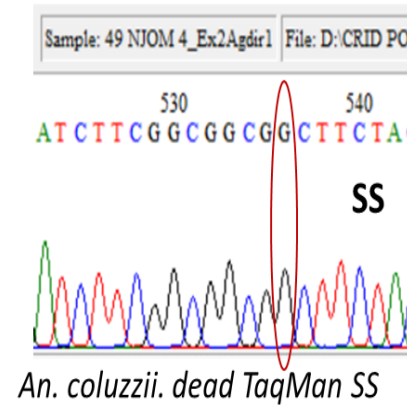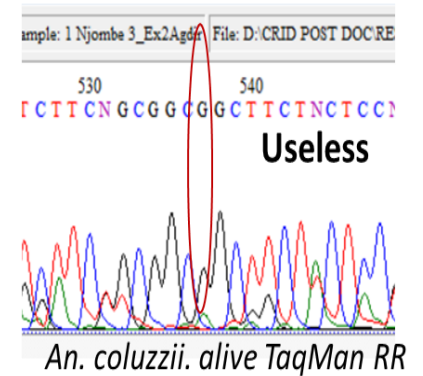

Supplement: S5 Fig — In An. gambiae s.s. (a) and An. coluzzii (b) from Njombe, relative to his phenotype and TaqMan genotype. An. gambiae s.s. alive RR and An. coluzzii dead RS have the A pick for resistant allele. (PDF) [file pone.0321825.s005.pdf]
