# Supplementary material for: Susceptibility profile of Anopheles and target site resistance mechanism against organophosphates in Cameroon
Source: PLoS One. 2025 May 22;20(5):e0321825. doi: 10.1371/journal.pone.0321825 (PMC12097638; doi:10.1371/journal.pone.0321825)
Supplement: S3 Fig — (a) 5 sequences of dead individuals that were genotyped as RR with the TaqMan, but present on the 3 readable sequences with two overlapping picks of A and G (b) 5 sequences of dead individual SS TaqMan genotype, presenting one pick of G on the 4 readable sequences. Useless sequences are those that exhibit multiple, overlapping peaks throughout the chromatogram. This overlap makes it difficult to confidently distinguish true heterozygotes from non-heterozygotes. They were then discarded. (PDF) [file pone.0321825.s003.pdf]

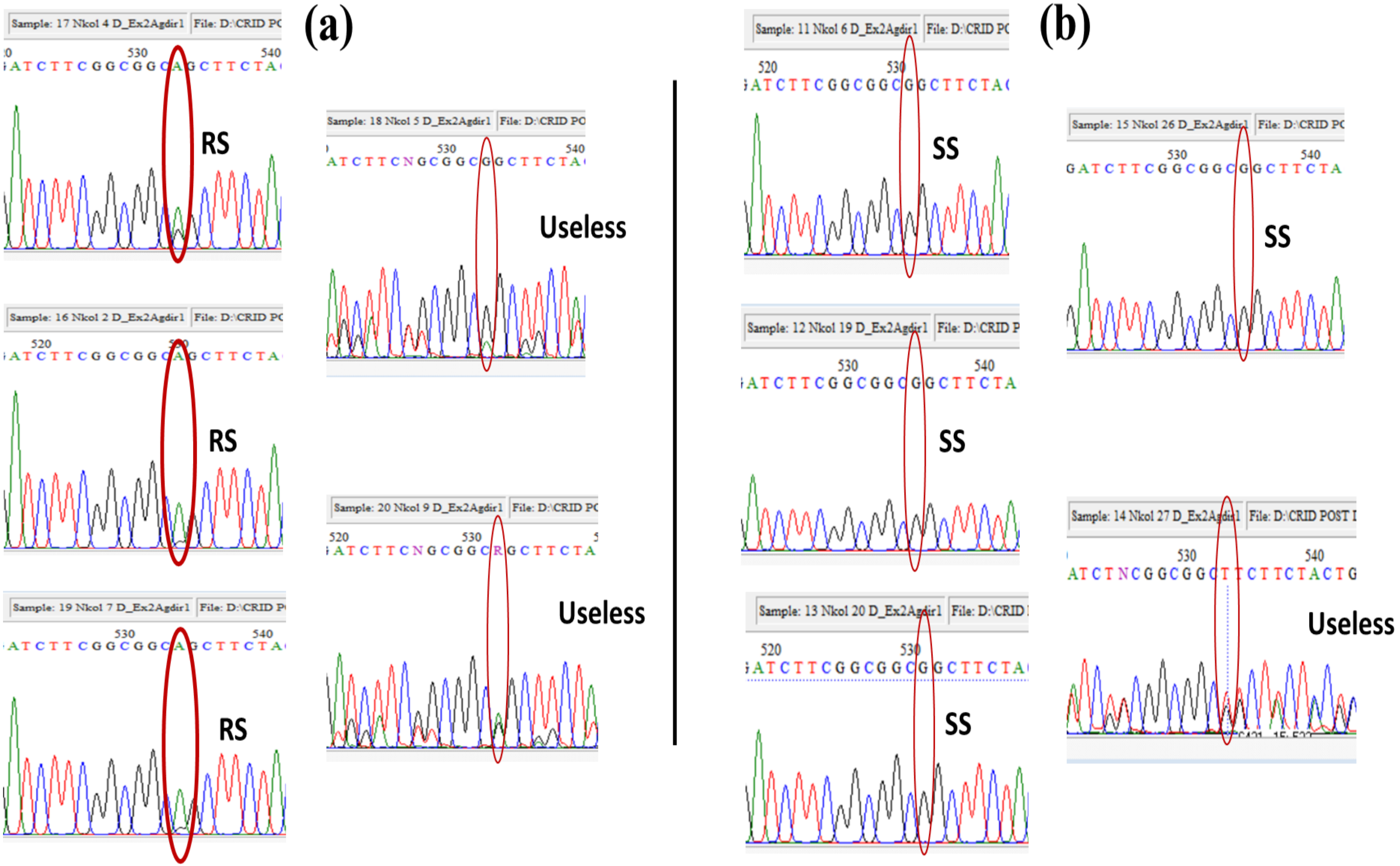

Dead TaqMan "RR" individuals

Dead TaqMan "SS" individuals
