# Supplementary material for: Susceptibility profile of Anopheles and target site resistance mechanism against organophosphates in Cameroon
Source: PLoS One. 2025 May 22;20(5):e0321825. doi: 10.1371/journal.pone.0321825 (PMC12097638; doi:10.1371/journal.pone.0321825)
Supplement: Attachment — Submitted filename: My Review for PONE-D-24-54840.docx [file pone.0321825.s006.docx]

**Manuscript PONE-D-24-54840**

The manuscript provided an insight into a countrywide resistance profile to organophosphates of two main malaria vectors in Cameroon and the related target site mechanism involved. Overall, the paper is relevant in its field and shows prospectives for decision making on organophosphates use in a context of pyrethroid resistance management. The methodology is in line with standards and the data generated was analysed in an appropriate way. The outcomes were discussed alongside with recent other studies from African malaria endemic settings.

**Comments/Recommendations/Questions**

Throughout the document: Replace “*An. gambiae”* by “*An. gambiae s.s.”* (when it is not referring to the complex). The use of “*An. gambiae s.l.”* is appropriate only while referring to the complex of species.

Line 30: Replace “WHO assays” by “WHO tube bioassays”

Line 40: Remove “moderately” as no resistance intensity bioassays were conducted against the mosquito population assessed.

Line 103-107: It looks like authors were highlighting the outcomes of the study. Better remove or rephrase as this is not the very best place for it.

Line 112: Replace “Anopheles” by “*Anopheles”*

Line 123-124: Replace "to insecticides” by “to the discriminating concentration of insecticides”.

Line 130: Replace “impregnated papers 0.25% PM,131 5% MA,” by “impregnated papers at discriminating concentration (0.25% PM, 5% MA)”

Line 132: The RH conditions used by authors overlap with the WHO-recommended range of 75% ± 10%, unlike temperature during which is supposed to be at 27 °C ± 2 °C (25-29°C). The 25 ± 1°C temperature slightly looked out of the WHO requirements.

Line 212-216: It is not clear whether species identification was performed on the whole mosquito size or a sub-sample. Kindly clarify in the manuscript.

Line 219: The number of mosquito specimens tested per location is not provided. It might be interesting to do add this on the graphs.

Line 224: At Njombe where vector population is composed of a mixture of *An. colluzzii* and *An. gambiae ss*, it would be preferable to present the dater per species.

Line 225: Replace “moderate resistance” by “resistance” (because no resistance intensity assay was conducted).

Line 231-236: Put in italics

Line 248: The way the pie charts are displayed around the map on Figure 2 requires improvement to avoid any sort of confusion. Better to have the stars replaced by the pie charts inside the map or not having a map at all with legend clear showing to what areas belongs the data.

Line 252-253: Put in italics

Line 256-258: Express the genotype in terms of proportion rather than number (61 individuals, 10 individuals, etc…)

Line 273-275: Express the genotype in terms of proportion as requested above.

Line 276: Replace “Ace” by “Ace-1”

Line 277-278: Put in italics

Line 283-285: Is there any justification of the mortality/genotype-based sampling used for Ace-1 gene polymorphism analysis? Same question for the sample size.

Line 334-338: Put in italics

Line 367-369: Put in italics

Line 382-384: Put in italics

Line 389: Replace “effectiveness” by “susceptibility”

Line 552-558: Reference number 10 is incomplete.
