# Supplementary material for: Susceptibility profile of Anopheles and target site resistance mechanism against organophosphates in Cameroon
Source: PLoS One. 2025 May 22;20(5):e0321825. doi: 10.1371/journal.pone.0321825 (PMC12097638; doi:10.1371/journal.pone.0321825)
Supplement: Attachment — Submitted filename: Respond to reviewers_Dandi-Labou et al.docx [file pone.0321825.s008.docx]

**Corresponding author:** Judith Dandi-Labou

**Address:** Centre for Research in Infectious Diseases (CRID),

P.O. BOX 13591 Yaoundé, Cameroon

**E-mail :** judith.labou@crid‑cam.net

**Tel.:** (237) 677 70 44 01

Monday, 10^th^ February 2025

**Cover letter for manuscript re-submission**

**To the Editor in Chief of *PLOS ONE***

Dear editor,

Thank you for your time and consideration of our manuscript entitled “Nationwide susceptibility profile of *Anopheles* and target site resistance mechanism against organophosphate in Cameroon**”,** which was recently submitted to your journal. We are grateful to the reviewers for their thoughtful comments, which have significantly strengthened the manuscript.

We have carefully addressed their feedback and incorporated revisions within the text. As a major change, we have modified the title of the manuscript to suit our findings as suggested by the reviewer. New title: “Susceptibility profile of *Anopheles* and target site resistance mechanism against organophosphates in Cameroon.” Moreover, we have reproduced the two figures that necessitated to be refined. QGIS version 3.28.3 was used to generate the map using open access share files (https://gadm.org/). The manuscript has also been formatted according to PLOS ONS’s requirements: affiliations and levels heading have been formatted according to recommendations, and funding and competing interest information have been removed.

We are confident that the revised manuscript effectively addresses all feedback and now meets the rigorous quality standards for publication in *PLOS ONE*

Thank you again for your time and consideration. We look forward to hearing from you soon.

Sincerely,

**Corresponding author**

Judith Dandi-Labou

Reviewer comments to the Author

Reviewer #1: 
Comments/Recommendations/Questions
Throughout the document: Replace “*An. gambiae*” by “*An. gambiae s.s*.” (when it is not referring to the complex). The use of “*An. gambiae* s.l.” is appropriate only while referring to the complex of species.

Thank you. *An. gambiae* has been replaced by *An. gambiae s.s.* when not referring to the complex throughout the document as suggested.

Line 39: Replace “WHO assays” by “WHO tube bioassays”

Ok, replaced. See line 39

Line 40: Remove “moderately” as no resistance intensity bioassays were conducted against the mosquito population assessed.
Ok, we have changed with “potentially resistant”. Lines 40- 41

Line 103-107: It looks like authors were highlighting the outcomes of the study. Better remove or rephrase as this is not the very best place for it.
OK, the sentence has been removed and sent to the conclusion section Lines 530-534

Line 112: Replace “Anopheles” by “*Anopheles*”
Ok. The word *Anopheles* has been italicized. Line 113

Line 123-124: Replace "to insecticides” by “to the discriminating concentration of insecticides”.
Ok. Replaced. Lines 133-134

Line 130: Replace “impregnated papers 0.25% PM,131 5% MA,” by “impregnated papers at discriminating concentration (0.25% PM, 5% MA)”
Ok. Replaced. Lines 140-141

Line 132: The RH conditions used by authors overlap with the WHO-recommended range of 75% ± 10%, unlike temperature during which is supposed to be at 27 °C ± 2 °C (25-29°C). The 25 ± 1°C temperature slightly looked out of the WHO requirements.

The reviewer is absolutely correct. During our bioassays, we recorded temperatures ranging from 24°C to 26°C, with an average of 25°C. While we did not observe any mortality in our control groups from the *An. funestus* s.s. population whose temperature was conducted at 24 °C, we acknowledge the importance of adhering strictly to WHO temperature recommendations. We appreciate your feedback and will ensure greater rigor in temperature conditions in future studies, in line with WHO guidelines.

The word “standard” have been removed. Line 142

Line 212-216: It is not clear whether species identification was performed on the whole mosquito size or a sub-sample. Kindly clarify in the manuscript.
Line 235-238

For populations known from previous studies to have only one primary vector species, we identified the species of just a subset of mosquitoes. However, in areas where multiple vector species are known to coexist (sympatric species), we performed species identification on all individual mosquitoes exposed to the insecticide.

We have clarified this in Material and Methods section. DNA extraction and species identification. Lines 151-156

Line 219: The number of mosquito specimens tested per location is not provided. It might be interesting to do add this on the graphs.
We tested as recommended by WHO (4 replicates of 25 mosquitoes each) 100 mosquitoes per location per insecticide, having 6 locations and two insecticides this represented 200 mosquitoes tested in each location, giving a total number 1200 adult females mentioned in line 243. Since this was not explicitly stated, we added the requested information in the manuscript line 243

Line 224: At Njombe where vector population is composed of a mixture of An. colluzzii and An. gambiae ss, it would be preferable to present the dater per species.
At Njombe, 10% of the 100 mosquitoes tested belonged to *An gambiae* s.s and 90% to *An coluzzi*, as mentioned in lines 238-240. *An gambiae* s.s. were few, and our targeted species were *An. coluzzii*. We removed them from the bioassay result

Line 225: Replace “moderate resistance” by “resistance” (because no resistance intensity assay was conducted).
Ok. “Moderate resistance” has been replaced by “possible resistance” following WHO recommendation. Line 250

Line 231-236: Put in italics
Lines 256-261 According to figure caption formatting, the legend is not in italics

<https://journals.plos.org/plosone/s/figures>

Line 248: The way the pie charts are displayed around the map on Figure 2 requires improvement to avoid any sort of confusion. Better to have the stars replaced by the pie charts inside the map or not having a map at all with legend clear showing to what areas belongs the data.
Ok, the figure has been reproduced. See Figure 2

Line 252-253: Put in italics
Lines 276-277 According to figure caption formatting, the legend is not in italics

<https://journals.plos.org/plosone/s/figures>

Line 256-258: Express the genotype in terms of proportion rather than number (61 individuals, 10 individuals, etc…)
OK. Proportions are added in brackets for more clarity. Lines 280-292

Line 273-275: Express the genotype in terms of proportion as requested above.
OK. Proportions are added in brackets for more clarity. Line 299-300

Line 276: Replace “Ace” by “Ace-1”
ok replaced Line 302

Line 277-278: Put in italics
Line 303-304 According to figure caption formatting, the legend is not in italics

<https://journals.plos.org/plosone/s/figures>

Line 283-285: Is there any justification of the mortality/genotype-based sampling used for Ace-1 gene polymorphism analysis? Same question for the sample size.
(Line 308-310) For cost purposes, we decided to confirm the genotyping on 20 individuals over 71 genotyped. Among the 20, we took 10 Alive and 10 dead Mosquitoes.

Being intrigued by the fact that many mosquitoes that were TaqMan RR died, we then divided the 10 dead among the two TaqMan genotypes we had (5 TaqMan RR and 5 TaqMan SS). The sequencing revealed that those “dead TaqMan RR” mosquitoes were heterozygotes. Having those sequences, we performed then the polymorphism analysis for a deeper understanding.

Line 334-338: Put in italics
Line 361-365 According to figure caption formatting, the legend is not in italics

<https://journals.plos.org/plosone/s/figures>

Line 367-369: Put in italics
Line 393-395 According to figure caption formatting, the legend is not in italics

<https://journals.plos.org/plosone/s/figures>

Line 382-384: Put in italics
Line 408-410 According to figure caption formatting, the legend is not in italics

<https://journals.plos.org/plosone/s/figures>

Line 389: Replace “effectiveness” by “susceptibility”

415-416 Since we are talking about the efficacity of the insecticide we think the word “effectiveness” is more appropriate than “susceptibility” which refers in this sentence to the response of the mosquitoes rather than to the action of the insecticide

Line 552-558: Reference number 10 is incomplete.

OK this have been adjusted. Line 596-597

USAID/President’s Malaria Initiative. Malaria Operational Plan FY 2018 and FY 2019; USAID/PMI:Cameroon, 2018.

Reviewer #2: 
Title
The authors may consider rephrasing the manuscript’s title to reflect its content as “Susceptibility profiles and target site resistance mechanisms of Anopheles malaria vectors to organophosphates in Cameroon.” Although the study was carried out in six localities across five regions of Cameroon (5/10 regions), the volume of data produced in this manuscript does not support extrapolation of these findings to the whole country, and in some study sites as well. For instance, from the six targeted localities, only 20 mosquitoes were sequenced to assess the genetic diversity of the Ace1 gene across the sites, which is statistically not accurate.

The reviewer has raised a valid point about the limitations of extrapolating our findings to the entire country based on the current dataset. We agree that a more precise title would better reflect the content of our study.

The revised title now reads: " Susceptibility profile of *Anopheles* and target site resistance mechanism against organophosphates in Cameroon." Line 2

Abstract
Line 32-33: replace “leading to…” with “necessitating the…”. The use of alternative insecticides due to escalating pyrethroid resistance is a necessity, rather than just a consequence of it.

Ok changed see line 32

Line 35: replace “such” with “these”

Ok changed see line 35

Line 36: replace “…resistance against…” with “…resistance to…”
Ok changed see line 36

Line 41: The sentence should be closed with a period/dot before starting the next sentence with "In contrast". If the use of the conjunction “both” refers to Anopheles gambiae s.s. and An. coluzzii, please specify this in the sentence, as in the previous sentence you talked about An. funestus and An. coluzzii instead.

In contrast of the two *Anopheles coluzzii* populations, - where one was fully susceptible (the population from north) and the second displayed a possible resistance (the population from the south);- the two populations of *An. gambiae* ss of our study were resistant.

So we used “both” to mention the two *An. gambiae* s.s. populations (Mangoum and Nkolondom).

We have rephrased, changing “both” with “the two *An. gambiae s.s*. populations in this study were resistant…” see lines 41-45

Line 42: please define the meaning of PM and MA in the previous sentences (may be in line 36) where the full names of related insecticides are provided for the first time in the text.
Ok; done. See lines 36-37

Line 42: in the peri-urban. To link many ideas in the same sentence, commas should be added after the words Nkolondom and MA (46% mortality).
Ok, comma added. See lines 43 and 44

Line 45: replace “alignment’ with “association” and add a comma after the word profile.
Ok, replaced. See line 46

Line 46: rephrase this sentence as … in the northern population of An. coluzzii, and …
Ok, “the” has been added. See line 47

Line 47: a higher frequency and not higher frequency
Ok, “a” has been added. See line 48

Line 50: add a comma after the word resistance
Ok, added. See line 50

Lines 54-55: replace but with however and rephrase the sentence as follow: However, it will be necessary to consider the distribution of species and the development of resistance.
Ok, replaced and the sentence has been rephrased. See lines 53-54

Introduction
Line 59-61: please use the new the recent world malaria report 2024 from the WHO to update the malaria burden data provided.
Ok, the reference has been updated. See line 59-62

Line 63: IRS is not a tool. Replace “tools” by “intervention strategies”
Ok, replaced. See line 64

Line 64-66: During the past decades, malaria control interventions have relied on four main classes of insecticides: organochlorines (since 1940s and 1950s for DDT), pyrethroids (since 1790s), organophosphates (sine 1960s), and carbamates (since 1970s) [ https://malariajournal.biomedcentral.com/articles/10.1186/s12936-018-2244-2 ].
Line 64-67. Unfortunately except the DDT, we have not been able to find in the proposed article the other insecticide and date related so we didn’t totally rephrase as proposed by the reviewer Line 65

Line 64: remove “their” before widespread.
the sentence has been modified

- from “While pyrethroids currently dominate in LLINs due to their high mosquito killing efficacy and low mammalian toxicity, **their** widespread resistance among vectors poses a growing challenge to malaria control”
- To: ”While pyrethroids currently dominate in LLINs due to their high mosquito killing efficacy and low mammalian toxicity, **the** widespread resistance **of mosquito vectors to this class of insecticide** poses a growing challenge to malaria control”. Line 68-69

Line 74: concerns have, not has
Ok. Corrected. Line 77

Line 79-80: With the cross-resistance mechanism existing between carbamates (CX) and organophosphates (OP), how can one associate the observed Ace-1 mutation specifically with OP and not CX? Differentiating between these is crucial for validating one of the insecticide classes for the development of control tools, such as IRS-based OP utilization.

Line 80-81.

A way to associate the observed Ace-1 mutation to OP and not CX, could be to conduct bioassays with both carbamates and organophosphates on the same mosquito population and performing correlation tests between the presence of Ace-1R and the ability of mosquitoes to survive insecticide exposure. But in general the correlation is stronger in mosquitoes exposed to carbamates than to organophosphates. Ahoua Alou et al. (2010) discuss about reasons in their article titled ‘’Distribution of ace-1R and resistance to carbamates and organophosphates in *Anopheles gambiae s.s*. populations from Côte d'Ivoire.’’ (<https://link.springer.com/article/10.1186/1475-2875-9-167>), highlighting that it is a difficult question to address since many other parameters can be involved.

It should be noted that insecticide resistance is dynamic and complex, particularly regarding Ace-1, which exhibits copy number variations (CNVs). This resistance even varies among insecticides within the same class, as shown in this article, where the association of Ace-1R is stronger with Malathion than with Pirimiphos-methyl. One hypothesis arising from this work is that to survive Pirimiphos-methyl, mosquitoes might possess multiple well-conserved copies of the resistant allele. However, similar research needs to be conducted on mosquitoes exposed to Malathion for more accurate conclusions, since this article focused mainly on mosquitoes exposed to Pirimiphos-methyl to address CNV.

Lines 96-99: …However, with the increasing use of OPs in agricultural settings, it is imperative to continuously monitor the presence and the extent of mosquitoes’ organophosphates resistance in the field, especially insecticides recommended by WHO for IRS.

What is the extent of pirimiphos-methyl-based insecticide usage in agricultural settings in Cameroon, and how does this vary across the different malaria-endemic epidemiological zones?

Can the authors provide detailed information on the use of pirimiphos-methyl-related insecticides in Cameroon, as this is crucial for understanding the distribution of resistance alleles across the country or the targeted study sites?

We thank the reviewer for these questions which have substantially be addressed in the discussion, section entitled “3-Agricultural practices are key drivers of pirimiphos methyl resistance in *An. gambiae* “ Lines 470-477

Also, with the increased resistance to pirimiphos-methyl as reported by the authors (lines 91-92) in various countries implementing IRS, what other insecticides could be used for IRS, if this strategy is indeed very efficient in controlling mosquito vector expansion in endemic areas?

These details should be provided for better understanding of the data reported in this manuscript.

Some new classes of insecticides are positioned as valuable IRS insecticides against malaria vectors., Neonicotinoid (Acetamiprid, Clothianidin), Pyrroles (Chlorphenapyr) or Metadiamide (Broflanilide). But as proposed by WHO those insecticides, as well as PM, should be used in combination or in rotation with other methods or other insecticide classes as we said in introduction Lines 74-75

Material and Methods
Mosquitoes sampling

Lines 114-118: is the lack of immature stages of Anopheles gambiae not due to the period of the study and/or site characteristics (market farming practices for instance)? When was the study carried out? The authors should provide details on the study period and the characteristics of the targeted sites, which are crucial for understanding the choice of each sampling technique used. Additionally, given the emphasis on the contribution of agricultural practices to organophosphate (OP) cross-resistance in public health (Anopheles), providing more details on the agricultural practices and main vector distribution in each locality could help better understand the data produced in this manuscript.

114-117. The reviewer raises a valid point regarding the sampling period and the characteristics of the targeted site. However, previous studies have consistently reported full susceptibility to organophosphates of the same *Anopheles* species as from our study from Elende (Nkemngo et al., 2020), Mibellon (Menze et al., 2018), and Gounougou (Fadel et al., 2019) across different sampling periods. Therefore, we considered emphasizing the sampling periods unnecessary, though we acknowledge the potential impact of seasonality by specifying the month and year of collection (See Figure 1B). Furthermore, the particular agricultural practices at Nkolondom and Mangoum over the year, and their potential influence on resistance, are thoroughly addressed in the discussion section titled "3-Agricultural practices are key drivers of pirimiphos methyl resistance in *An. gambiae*." Line 470-480

TaqMan genotyping
Lines 147-148: correct this sentence as …each reaction was conducted in 10ul total volume containing….

OK corrected Line 163-164

What QC measures were taken to validate the qPCR results?
Quality control steps were implemented throughout the qPCR workflow, from experimental design to data analysis, to ensure trustworthy results. As we designed ourselves primers for qPCR, we assessed Standard curves and had good efficiency. We checked the stability of our chosen housekeeping gene across all experimental groups by ensuring similar Ct values. The Negative Control Samples were used to evaluate nonspecific gene amplification and contamination of reaction solutions.

Ace-a amplification and sequencing

Lines 165-166: From the 20 mosquitoes selected, how many were exposed dead and exposed survivors? Are the 20 mosquitoes from the same site or different sites? The authors should specify and provide more details on this. Finally, 20 mosquitoes seem not enough to validate the polymorphism of the Ace-1 gene in the mosquitos’ populations from up to 6 study sites. The authors should justify why only 20 mosquitoes sequenced, and raise this concern as a limitation to this study, as the observed genomic variations could not be extrapolated nationwide.

The reviewer has raised a valid point here, however the main goal of sequencing was first of all to confirm the genotyping results and not to assess polymorphism. All the 20 mosquitoes were from Nkolondom. this information was provided in results section (Ace-1 gene polymorphism analysis- Confirmation of genotyping result by direct sequencing) Line 308-311. But for clarity we corrected by adding the precision of the site (locality) in Materials and methods (See line 182)

From the 20 mosquitoes selected, 10 were exposed dead and 10 were exposed survivors.

Since one of the objectives was to confirm by sequencing the genotypes obtained using the TaqMan machine, among the 10 exposed dead, 5 were dead TaqMan-RR and 5 dead TaqMan-SS.

These precisions were given in results section (Ace-1 gene polymorphism analysis-

Confirmation of genotyping result by direct sequencing) as we think that it is the most suitable place to give those details Line 308-317.

As previously stated, the 20 mosquitoes were exclusively from Nkolondom exposed to PM. We evaluated the resistance profile and genotyped the Ace-1 gene across all six locations. Only *Anopheles gambiae* s.s. from Nkolondom exhibited clear resistance to both PM (80% mortality) and MA (46% mortality) making samples from this locality more suitable for the Ace-1 polymorphism analysis. Due to the prioritization of funds, we chose to focus initially on mosquitoes exposed to PM rather than those exposed to MA, as PM is the “newest” insecticide recommended by WHO.

Line 168: rephrase as … a 50ul reaction mix containing 10pml of each primer …
OK Modified Line 185

Line 173-174: rephrase as … visualized on 1.5% agarose gel stained with Midori green.
OK Modified Lines 190-191

Line 181: using default parameters of Tamura 3 model.
OK Modified Line 198

Line 182: …was built in network software. Specify the version of this online-based software.
OK. The version has been added Lines 199-200

Line 199: close the first brackets opened.
OK, the bracket in the middle has been removed Line 220

Lines 202-204: rephrase this sentence to complete the missing word. Each reaction mixture contained 1ul genomic DNA, 0.6uM of …..

OK Modified Lines 223-224

Was the study not subjected to ethical and/or administrative considerations? The authors should clarify on this in a short subsection.

This study is a part of a project that received ethical clearance from the National Research Ethics Committee for Human Health, registered under number 2021/07/1372/CE/CNERSH/SP to ensure the safety of study participants during the collection of adult mosquitoes. Furthermore, permissions were obtained from village chiefs and homeowners at the locations where mosquitoes were collected.

Results

Line 213: An. funestus constituted the main vector species in Elende and Mibellon, while An gambiae s.s constituted the main species in Mangoum and Nkolondom.
OK Modified Line 235-237

Lines 226-227: rephrase as: Alarmingly, mosquitos’ populations from Nkolondom exhibited greater resistance level to PM (80% mortality) and 46% (mortality).
OK Rephrased Line 251-253

Line 246: …in all An. funestus populations, supporting….
OK. “the” has been removed Line 270

Line 256: From the XX individuals exposed to PM in Nkolondom, 61 exhibited the homozygous resistant allele RR, 10 exhibited the homozygous susceptible allele SS, and no heterozygous genotype (RS) was found in this population.
OK Modified Lines 280-284

Line 258: if only 30/61 RR mosquitoes were alive, what was the status of the remaining 31 mosquitoes? if they were dead mosquitoes, could we infer that the distribution of the RR genotype is similar between alive and survivors’ mosquitoes from Nkolondom? Hence, to what extend could we associate the survivor status of these mosquitoes populations with the presence of the R allele?
Line 290. The 31 remaining mosquitoes were dead.

For the TaqMan genotyping of 30 RR survivor mosquitoes and 31 RR dead mosquitoes, we observed that the distribution appeared similar between the two groups. However, all surviving mosquitoes were RR (This translates to a 100% frequency of the Ace-1R allele among surviving individuals), whereas this was not the case for the dead mosquitoes (all SS mosquitoes were found exclusively among the dead group 10/41). This discrepancy resulted in a slightly low but still significant P-value for the correlation between RR and SS (OR = 20.33, 95% CI = 1.14–362.38; P < 0.04).

We have added for your reference a screenshot of re-analyzed correlation test

Lines 260-261: It is written that “Mosquitoes with Ace-1R allele displayed a significantly higher ability to survive compared to those with the Ace-1S allele (OR=64.37, 95% CI=3.85–1075.38; P < 0.003)” – However, This seems not true if dead mosquitoes also carry the R allele at >50% (31/61) of the genotyped populations (see comment above, line 258).

Line 285-289. The ability to survive is compared between Ace-1R and Ace-1S allele. This late (Ace-1S) is not found among survivors, even if all mosquitoes having R allele are not surviving.( That is the reason why we went further first to confirm the genotype by sequencing, then to carry out CNV analysis. To realized that all the dead TaqManRR were dead RS, all the dead TaqManSS remain dead SS )

Although dead mosquitoes carry the R allele at 50%, we should not neglect the fact that all S alleles were amount dead and that all alive mosquitoes were R. Those two main parameters have a wide impact on the correlation.

Line 281: the author should consider removing the subtitle ‘Confirmation of genotyping result by direct sequencing’ from the text. The main title (Ace-1 gene polymorphism analysis) already explains the main idea of the following paragraph.
Line 307. This subtitle (Confirmation of genotyping result by direct sequencing) refers to the comparison conducted on the same samples using both TaqMan genotyping and direct sequencing. This comparison helps to determine the accuracy of TaqMan genotyping results. It does not include additional analyses of polymorphisms, such as diversity indices or phylogenetic analysis, which are covered in other section within the main title (Ace-1 polymorphism analysis)

Lines 282-283: the authors should rephrase this sentence to correct grammatical and vocabulary errors, and fill in the missing words.
Ok rephrased Lines 308-309

Line 289: two sequences and not two sequencing.
Ok Corrected. Line 315

Line 298: Why choosing only 6 mosquitoes for sequencing from this site, giving that bioassays in this site were performed against MA and OP.

Lines 324-327. Since the population from Njombe was fully susceptible to PM but showed signs of resistance to MA, we were interested in investigating whether the surviving mosquitoes carried the Ace-1R allele.

For precision, after the WHO bioassay test, three mosquitoes were still alive. Upon species identification, we found that two were *Anopheles gambiae* s.s. and one was *Anopheles coluzzii*. Since our target species in that locality was *An. coluzzii*, we excluded *An. gambiae* from the results to avoid bias.

Nevertheless, we genotyped all individuals and found that the three surviving mosquitoes were TaqMan RR, one dead was RS (*An. coluzzii)*, and the rest of dead mosquitoes were SS. We further sequenced those TaqMan genotypes and confirmed the genotype and the correlation between the R allele and survival phenotype. This result underscores the urgent need for monitoring the evolution of this marker in this locality.

Line 344: Table presents…
Ok Corrected. Line 371

Line 351: … the cloning helps…
Ok Corrected Line 378

Lines 371-379: How is the CNV data related to the genetic diversity of the Ace1 gene? Giving that the genetic diversity was higher in exposed dead mosquitoes, while the CNV is higher in exposed survivor mosquitoes.
Line 399-346. This result has been discussed in the discussion section (4 - Duplication of Ace-1 worsens resistance to OP in *An. gambiae* s.s. in Cameroon), lines 494-519.

These contrasting results lead us to the hypothesis that for mosquitoes to survive PM, they must possess multiple identical (monomorphic) copies of the Ace-R alleles. This could explain why the RS and SS mosquitoes died. However, further studies need to be conducted to draw definitive conclusions

Discussion
Line 430: Our and not your.
Ok Corrected Line 457

Lines 432-432: it is read that “The 3% frequency of the Ace-1 allele in An. coluzzii population from Njombe suggests a recent emergence of the mutation in this species in Cameroon” -
We thanks the reviewer for pointing this out. The sentence has been rephrased. This low mutation frequency might suggest a recent emergence of this mutation not in Cameroon, but in the locality. Correction have been made. See Lines 459-463

Line 456-457: The authors should explain the significance of the Tajima's and Fu's 457 & Li's indexes for better understanding of these data. Moreover, I am afraid that the number of samples sequenced (<20 sequences analyzed) in this study are not enough for a statistical validation of the data and to support the conclusions raised by the authors. The authors should be cautious is reporting such limited data. I will advice to mostly emphasize on the presence or not of the mutations in the study sites, and avoid any extrapolation that could bias the extend of the data to the entire study site or region or country.

Lines 485-486 A negative Tajima's D value suggests there could have been a selective sweep, indicating that the population has experienced a reduction in genetic diversity due to positive selection. That can explain the low diversities indices. Similarly, a negative Fu’s & Li’s value indicates an excess of low-frequency alleles, which is consistent with recent selective sweeps. However, It can also suggest population expansion

A summarized explanation has been added as proposed by the reviewer. see Lines 486-487

The 20 sequences (out of the 71 individuals genotyped) were obtained from both live and dead mosquitoes exposed to pirimiphos-methyl (PM) in the same locality, Nkolondom (as explained earlier). The results from the 100 cloned sequences derived from these individuals enhance the reliability of this study, even with this small sample size. However, the reviewer is correct in noting that the number of sample sequences is insufficient to generalize the findings to the entire study. The title is revised based on the referee's suggestion, and this limitation has been acknowledged. Line 487

459-460: What could be the consequences of the greater haplotype and nucleotide diversity observed among exposed dead mosquitoes after exposure to insecticides (OP or CM) on the efficacy of the IRS-based OP? given that the exposed survivors seem less diversified. The authors should provide further details on this to support the use of OP for IRS interventions in malaria endemic areas in short and long terms.
Line 488-490. A more diverse population may be more susceptible to pirimiphos-methyl, which seems advantageous for vector control efforts. However, as the reviewer pointed out, drawing consequences about the efficacy of IRS-based interventions using organophosphates or carbamates requires further research due to the complexity associated with this resistance mechanism (Ace-1) and the involvement of other potential complementary mechanisms that may influence the observed resistance.

Line 462: the title reads “Duplication of Ace-1 worsens resistance to OP in An. gambiae in Cameroon” – However, sequencing of only five mosquitoes cannot generate enough data to support such conclusion. The authors should be modest in emphasizing about the strength of the data produced in their study. They should rephrase this title to report only the presence of duplicated copies of the Ace1 gene in the analyzed mosquitoes population (5) and recommend further investigations to assess it extend in a large mosquitoes populations from the same sites and other localities.
Thank you to the reviewer, the subtitle has been modified Line 492

Conclusion
From the findings of this study, the authors should consider reporting the resistance profile of targeted mosquito species against malathion and pirimiphos-methyl, as well as the presence or absence of Ace1 mutations in the mosquito populations circulating in the study sites. The data generated from sequencing and qPCR analysis of the Ace1 molecular target are not sufficient enough to draw confident statistical relationships about the phenotypic and genotypic resistance patterns of mosquito populations against malathion and pirimiphos-methyl in this study.

Recommendations have been taken into account. the conclusion has been modified Line 529-543

The conclusion highlights the heterogeneity in susceptibility profiles of the population in Cameroon and emphasizes the importance of monitoring mosquito populations before implementing IRS. It points out that some populations are already resistant to organophosphates and underscores the role of the Ace-1-resistant allele, further stressing the need for nationwide evaluation. Additionally, it acknowledges the potential involvement of other mechanisms, such as metabolic resistance that should be elucidated.

Reviewer #3:

Line 37 : ''...three malaria vectors (Anopheles gambiae s.s., An. coluzzii, and An. funestus s.l.)...''. I think the author should choose between the Anopheles complexes, in which case he replaces Anopheles gambiae s.s. and An. coluzzii with An. gambiae s.l. Otherwise, he should specify the molecular species within the An. funestus complex. Otherwise, it's confusing.

Same of line lines 100 and 101. You should update the whole document, because in the manuscript you write An. gambiae without specifying the species or whether it is the complex. I know that others often prefer An. gambiae to An. gambiae s.s., but you weren't precise at the beginning.

We are grateful to the reviewer for this comment. The correction has been made throughout the document. *An. funestus* s.s. and *An. gambiae* s.s. have been specified

Line 103-105: We revealed a... is this sentence necessary for this introductory section? it's not too clear and could be deleted or improved.

OK the sentence has been removed and sent to the conclusion section Lines 530-534

The author should ask a geographer to help him reproduce Figures 1 and 2. Otherwise these figures do not represent the work of a scientist.

OK Figure 1 and 2 have been reproduced as suggested. QGIS version 3.28.3 has been used to generate the map using open access sharefiles (https://gadm.org/)"

Line 110: The author must specify the data collection period

Since the collection period was presented in fig 1B and commented (lines 134-136) we didn’t judge important to precise again in the text

Line 114 : ...Indoor aspiration method on adults' mosquitoes was used... In this case, the age of the mosquitoes will not be known and you will have to specify this as a limitation of the study in the discussion.

Indeed, the age of mosquitoes that have been aspirated indoors is not known.

These adult mosquitoes collected by indoor aspiration were subjected to forced egg-laying and the resulting larvae were reared to F1 adults. Those are the adult F1 females that are mentioned in the sentence “Two- to five-day-old F0 or **F1** adult female mosquitoes…”.

We corrected this by adding clarifications in the manuscript. Line 118-121

Line 130 : Capture by Indoor aspiration method will not give you the age of the mosquitoes

The F0 comes from immature stages collection, and F1 is the progeny of the adult aspirated indoor (see previous comment)

Line 219: An. gambiae s.l. or An. gambiae s.s.? You need to be consistent, otherwise in ‘Species identification’ above you referred to An. gambiae s.s.

Modifications have been made within the text Line 243-253

As mentioned above, you should reproduce Figure 2

OK Figure 2 has been reproduced.

Lines 373-374 : Alive mosquitoes had a significantly higher number of Ace-1 gene copies (2.4 ± 0.5) Line 374 compared to dead SS... You should specify whether these living mosquitoes were RR or RS or both

Line 398-401. The mosquitoes used for qPCR are the same that were cloned to assess duplication. Since the genotypes are detailed in cloning section and in table 2 cited above, we judged not relevant to precise again.

Lines 387-388: I suggest that this 1st sentence of the discussion be deleted as it adds nothing to the discussion at this point

Lines 413-415. The reason of this sentence is to make the reader to remind the context of this work before discussion. Without offending the reviewer, we would like to keep this introductory sentence.

Line 430: ...was absent in ‘your’ study... or ‘our’ study... ? Please make sure you correct the sentence so that it is understandable

OK sentence corrected Line 457

Lines 432-435: 3% frequency and you're talking about emergence? I don't think so. Previous studies had shown 0% ? and by making a correlation you think that the difference is significant? If so, you should refer to studies carried out in the study area or in the country. It would be important to make corrections to this section

We suggest that the low frequency of Ace-1 detected at Njombe could be a sign of the emergence of this mutation not in Cameroon but in Njombe ( the correction has been made, Lines 459-460).

Although more investigation needs to be done, a study conducted in 2021 by Ngangue-Siewe et al 2022 in that same population (Njombe) shows the same pattern. The only survivor they have after exposition to Malathion had the Ace-1 resistant mutation. Moreover the allelic frequency of the Ace-1R in the unexposed population (composed of both An gambiae s.s. and An Coluzzii), was low 4.5%, contrasting with the neighbor *An. gambiae* populations with 41.1% (Kekem) and 87.9% (Santchou). https://doi.org/10.3390/pathogens11080824

Reviewer #4: Titre : Nationwide susceptibility profile of Anopheles and target site resistance mechanism against organophosphate in Cameroon.

Vectors are resistant to PM in several study sites.

1) Do you think it is appropriate to recommend this insecticide for IRS?

The duration of residual efficacy of insecticides applied to wall surfaces is one of the factors influencing the efficacy of indoor residual spraying. Several studies have shown that the remanence of PM is around 4 to 6 months (Chanda et al. 2013 in Zambia, Tchicaya et al., (2014) in Mbe in Côte d'Ivoire on banco and cement walls). On the other hand, a remanence of 8 months was observed by Haji et al. (2015).

PM can still be recommended despite its limitations:

-The resistance to PM is not widespread, many populations of malaria vectors are still susceptible as we have also shown in this paper.

-PM has been shown to be a good alternative in areas with widespread of pyrethroid resistance (Kane et al, 2020)

-A study conducted on IRS with Pirikool® 300CS (the new capsule suspension formulation of PM) showed high and prolonged mortality (10–12 months) of wild pyrethroid-resistant *An gambiae* sl. (Fongnikin et al 2022) https://doi.org/10.1371/journal.pone.0267229

2) Do you have any idea about the residual activity of PM in your context? If not, please specify the evaluation of residual activity of insecticides prior to the selection of IRS from the perspective of the study.

We don’t yet know the residual activity of PM in Cameroon, so the recommendation is added in the manuscript in conclusion part. Line 543-544

3) What is the duration of malaria transmission in the study area? Is it an area of perennial transmission? Mention the duration of transmission in the introduction.

If so, at least two rounds of IRS intervention per year are technically required. This requires considerable human and financial resources.

Cameroon has three malaria transmission seasons that vary by geographic region. The

forest zone in the south has a permanent transmission season of seven to twelve months. The North and Adamawa regions have a tropical climate but a shorter transmission season of four to six months. The Far North region has a tropical and Sahelian climate with a short seasonal transmission period of one to three months. (PMI Cameroon Malaria Operational Plan FY 2017)

However, after careful consideration, we believe that the focus of our study on the resistance mechanisms of mosquitoes to organophosphates is not directly influenced by this information on malaria transmission duration so we prefer not to include.

Overproduction of esterases has been associated with resistance to organophosphates and pyrethroids (Brogdon et al., 1999). It should be noted that increased expression of this enzyme may lead to detoxification of organophosphates.

Yes, this is why we mentioned in the discussion the potential impact of metabolic mechanisms and recommend in the conclusion section to investigate their contribution in our samples. (this work is ongoing)

4) Why haven't you done the enzyme test? If the results are available, include them in the results section of your study.

We are grateful, We are going to consider it in additional work to be done.

5) Do you think the Ace1 mutation is the only mechanism involved in the organophosphate resistance observed in Nkolondom?

No, we do not. Metabolic resistance mechanism could be involved as it has been found in other studies. RNA sequencing of those populations (both exposed to PM and to MA) has been later conducted, and analysis are ongoing

New generation insecticides such as SumiShield® 50WG and Fludora® Fusion, recently approved by the WHO, are currently available and could be used to cover the entire duration of malaria transmission in regions with perennial malaria (Agossa et al., 2018a; Agossa et al., 2018b).

6) How sensitive are vectors to these new insecticides? It is now inconceivable to carry out a study of vector resistance without including the new insecticides.

We acknowledge the importance of evaluating resistance to newer vector control products. While our study focused specifically on pirimiphos-methyl, we recognize the evolving landscape of vector control tools. Our colleagues in the institutions have recently published work on newer insecticides. We kindly refer the reviewer to the recent publications by Thiomela et al. 2024 (https://doi.org/10.1186/s12879-024-09630-4) and Tchouakui et al. 2024 (<https://doi.org/10.21203/rs.3.rs-3865779/v1>) for insights into vector sensitivity to these new products.
